# Simple molecular networks that respond optimally to time-periodic stimulation

**DOI:** 10.1186/1752-0509-3-29

**Published:** 2009-03-03

**Authors:** Axel Cournac, Jacques-Alexandre Sepulchre

**Affiliations:** 1 Institut Non Linéaire de Nice, Université de Nice Sophia-Antipolis, CNRS, Valbonne, France; 2 Laboratoire Matière et Systèmes Complexes, Université Diderot, Paris, France

## Abstract

**Background:**

Bacteria or cells receive many signals from their environment and from other organisms. In order to process this large amount of information, Systems Biology shows that a central role is played by regulatory networks composed of genes and proteins. The objective of this paper is to present and to discuss simple regulatory network motifs having the property to maximize their responses under time-periodic stimulations. In elucidating the mechanisms underlying these responses through simple networks the goal is to pinpoint general principles which optimize the oscillatory responses of molecular networks.

**Results:**

We took a look at basic network motifs studied in the literature such as the Incoherent Feedforward Loop (IFFL) or the interlerlocked negative feedback loop. The former is also generalized to a diamond pattern, with network components being either purely genetic or combining genetic and signaling pathways. Using standard mathematics and numerical simulations, we explain the types of responses exhibited by the IFFL with respect to a train of periodic pulses. We show that this system has a non-vanishing response only if the inter-pulse interval is above a threshold. A slight generalisation of the IFFL (the diamond) is shown to work as an ideal pass-band filter. We next show a mechanism by which average of oscillatory response can be maximized by bursting temporal patterns. Finally we study the interlerlocked negative feedback loop, i.e. a 2-gene motif forming a loop where the nodes respectively activate and repress each other, and show situations where this system possesses a resonance under periodic stimulation.

**Conclusion:**

We present several simple motif designs of molecular networks producing optimal output in response to periodic stimulations of the system. The identified mechanisms are simple and based on known network motifs in the literature, so that that they could be embodied in existing organisms, or easily implementable by means of synthetic biology. Moreover we show that these designs can be studied in different contexts of molecular biology, as for example in genetic networks or in signaling pathways.

## Background

For past several decades, the experimental data concerning protein concentrations in biological regulatory networks have mostly been obtained in conditions related to stationary states. In some natural contexts, however, or in some experiments, the relevant cellular response to measure is the one the cell displays when it is exposed to time-dependent signals. Such information can be essential to unravelling the regulatory principles of those molecular networks which by nature are repeatedly stimulated by time-varying inputs [1]. For example the gene expression within neuronal cells can be substantially affected by the time-dependent signals received from its afferent neurons, and this property is essential for the formation of memories [2]. Other examples can be met with in various contexts, like the frequency encoding phenomenon associated with Ca^2+ ^oscillations [3-6], or the response to time-dependent osmolarity shocks [7]. We note that, in these systems an optimal-response may exist with respect to periodic stimulations, meaning that for example, the mean production of some activated transcription factors, or of some proteins of interest, would be maximized if the external periodic signal follows some specific time-course and shape. This question has received little attention in the context of biological networks. This research topic is nevertheless timely, as recent developments of experimental techniques in molecular biology enables one to access time-dependent concentrations, and investigate new problems about the time-response of molecular networks.

From an experimental point of view, some interesting experiments were recently performed to reveal such time-response properties in biological systems [7-9]. In order to anticipate more experimental studies in this direction, the objective of this paper is to identify basic network topologies which allow for the property of optimal oscillatory responses in molecular biology. In the current literature only a small number of studies have tackled this issue and most of the articles addressing this question principally considers the linear response to sinusoidal perturbations. For example several papers demonstrate the low-pass filter property of small molecular networks [10-13]. Nearly all of these studies consider small amplitude perturbations, and use Fourier transform, discussing how the system parameters modify the cut-off frequency. As a matter of fact, when a system behaves like a low-pass filter, the frequency which maximizes the output response is 0. So in this case the system does not display a genuine temporal specificity. Very few studies have looked for the possibility of maximizing some variables at non-zero frequency in biological networks. A recent study [14], using again the tools of linearization and of Fourier analysis, shows that a signaling cascade with a negative feedback can behave as a band-pass filter, characterized by a frequency which maximizes the amplitude of the response. But when the system's nonlinearity is important, as is often the case in models of transcriptional regulatory networks, or when the system is linear but the periodic forcing is multiplicative (thus not additive), then Fourier decomposition of the input signal is no longer of general interest. Furthermore, in many instances, the time-dependent signals directed to regulatory networks are far from harmonic, they are often pulsatile. This is why a time-periodic input to molecular networks is often assumed as a periodic train of pulses. Smolen et al. studied a molecular network which was periodically stimulated by a train of spikes and which exhibited the ability to respond in a maximal fashion to a particular inter-spike interval [15]. Their model equations were nonlinear and associated with a lot of parameters, so the origin of the bandpass filter property is not straightforward. Another instance of periodic stimulations with a train of square pulses was thoroughly studied by Li and Goldbeter in the context of cellular receptor systems [16]. By using analytical calculations and numerical simulations of a linear model [17], they show that can exist an optimum stimulus pattern of periodic square pulses which maximizes a lumped observable. This is defined by the authors as the receptor activity. However, the Li and Goldbeter model has again many parameters, and the identification of the essential ingredients which enable one to induce a maximum amount of receptor activity is not obvious.

In the present paper we study simpler models, which pertain to basic network motifs found in biological regulations. We point out some principles which guarantee the existence of an optimal response in the production rates of output molecules, when these systems are activated by a periodic signal. The identified mechanisms are based on known network motifs in the literature and include, for example, the negative feedback loop and the incoherent feedforward loop (IFFL) motif studied by Alon and coworkers in the context of gene regulatory networks of bacteria [18]. Let us recall that in this framework a network motif is a small pattern of molecular interactions which recurs repeatedly in comparaison with what would occur in random networks. Here we will make use of this concept of motif in a broader sense, without requiring that its ubiquity be statistically proved, but requesting that it represents a basic network pattern associated with specific information-processing properties [19]. Moreover we will study these designs in at least two different contexts of molecular biology, namely in genetic regulatory networks or in signaling pathways. In the context of genetic regulation, the mathematical models are usually strongly nonlinear, involving sigmoidal regulation functions. In order to use analytical tools to study the behavior of these systems under time-dependent signals, we will employ the "logic" approximation, where regulation functions are replaced by all-or-none functions. In this case the nonlinear system is not approximated by a set of linear equations but by a set of piecewise-linear equations [20]. This approach has indeed been developed for many years in the study of genetic networks and has revealed to be quite fruitful [21].

## Results

### Simple models of biomolecule activations

In this Section, we give a brief summary of the simplest models used in the literature to describe activation processes, either at the gene level, or at the level of post-translational modification of proteins. We then report elementary but useful results which are obtained when the activation is implemented by means of a train of periodic square pulses.

**Figure 1:**
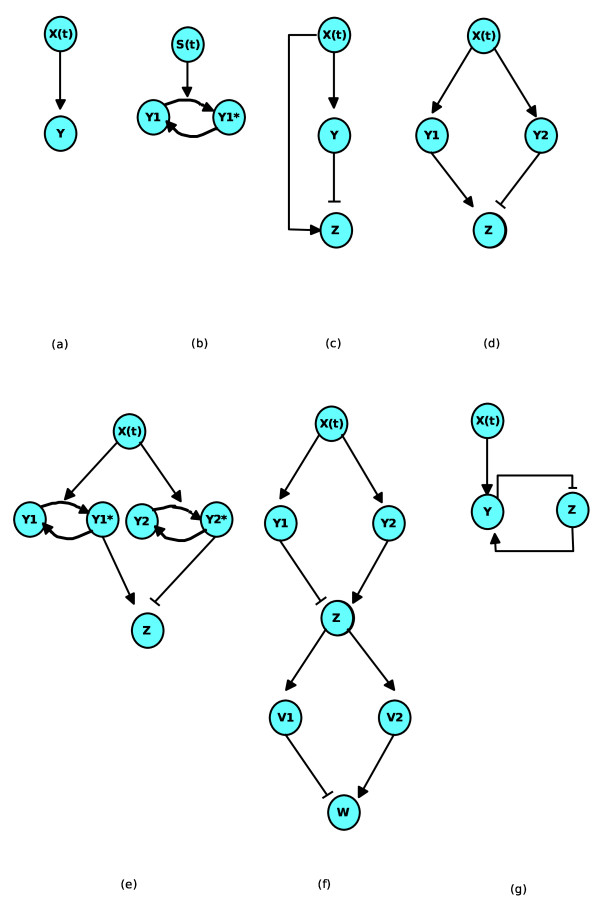
**Motifs of activations and regulations of small biomolecular networks**. (a) A simple activation (b) activation of a signaling cycle (c) the Incoherent Feed Forward Loop (d) the Diamond motif (e) Diamond motif with signaling cycles (f) two Diamond motifs associated in series (g) the interlocked negative feedback loop.

**Figure 2:**
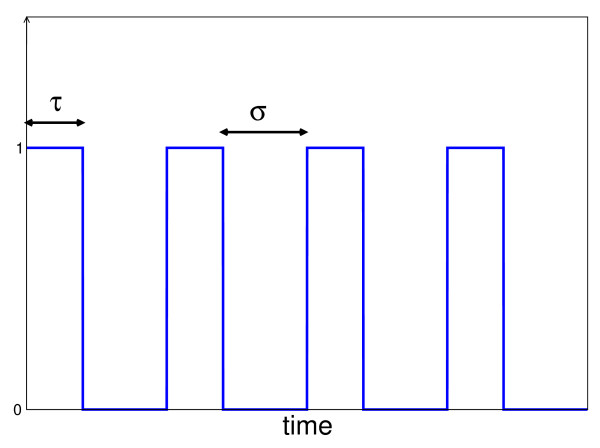
**Input stimulation signal**. The pulsatile signal is shown, the duration of the "on-phase" is denoted by *t*, the inter-pulse interval or the silent phase between the pulses, is denoted by *σ*. Thus the period of *S*(*t*) is given by *T *= *τ *+ *σ*.

Transcriptional regulatory networks can be described with genes being represented by letters *X*, *Y*, ... The same notation is used for the concentrations of the corresponding proteins synthesized by these genes. We first introduce what is called the "simple regulation" by Alon [18], a situation which is depicted in Fig. 1(a) by an arrow from *X *to *Y*. In this case the gene *Y *is positively regulated by only one transcription factor *X*, and the basic process of synthesis of protein *Y *from its gene is described by a single differential equation:

(1) $$\dot{Y}=\beta R(X)-\alpha Y$$

where *R*(*X*) is called the regulatory function. We consider regulatory functions bounded by 1. Typically *R*(*X*) has the form of a Hill function *X*^*n*^/(*X*^*n *^+ *θ*^*n*^) with some cooperativity *n *and an activation threshold *θ *but we will often use the *logic *approximation for *R*(*X*), where the latter is replaced by the unit step function *H*(*X *- *θ*) (with only two values *H*(*X*) = 0 if X *<*0, and *H*(*X*) = 1 otherwise). The parameters *β *and *α *are respectively the maximum synthesis rate of protein *Y *and the degradation parameter, including the possible dilution effect from cell growth. Whenever *R*(*X*) = 1 the system converges to a steady state *κ *= *β*/*α*. In the sequel *X*(*t*) is considered as a function of time, and one defines *S*(*t*) = *R*(*X*(*t*)). A class of signals *S*(*t*) which will be considered below is a periodic train of square pulses of amplitude 1, whose temporal pattern is characterized by the numbers (*τ*, *σ*) (cf. Fig. 2). The parameter *τ *describes the duration of the "on-phase" corresponding to the activation of transcription factor *X *binding gene *Y*. The inter-pulse interval, or the silent phase between the pulses, is denoted by *σ*. Thus the period of *S*(*t*) is given by *T *= *τ *+ *σ*.

In response to the input signal *S*(*t*), we will focus on three observables in this system, namely the extrema (minimum and maximum), and the average concentrations reached by *Y *(*t*), denoted respectively by *Y*_min_, *Y*_max _and ⟨*Y*⟩_*T*_. The extreme values of *Y *(*t*) can be the most relevant quantities in systems where *Y *is itself a transcription factor for other genes, because then what matters is the comparison of *Y *with a threshold of activation or of repression. On the other hand, if *Y *is consumed by some downstream process, then the mean concentration of *Y *(*t*) averaged over a period *T *of *S*(*t*), denoted by ⟨*Y*⟩_*T*_, is the relevant quantity since it quantifies the available protein synthesized over one period.

In the case of a simple regulation with periodic square pulses, the model equation (1) is easily worked out and from that calculation one deduces the observables ⟨*Y*⟩_*T*_, *Y*_max _and *Y*_min _as:

(2) $$\begin{array}{c} {\langle Y\rangle }_{T}=\kappa \frac{\tau }{\tau +\sigma }\\ Y_{\max}=\kappa \frac{1-e^{-\alpha \tau }}{1-e^{-\alpha (\tau +\sigma )}}\\ Y_{\min}=\kappa \frac{e^{\alpha \tau }-1}{e^{\alpha (\tau +\sigma )}-1} \end{array}$$

**Figure 3:**
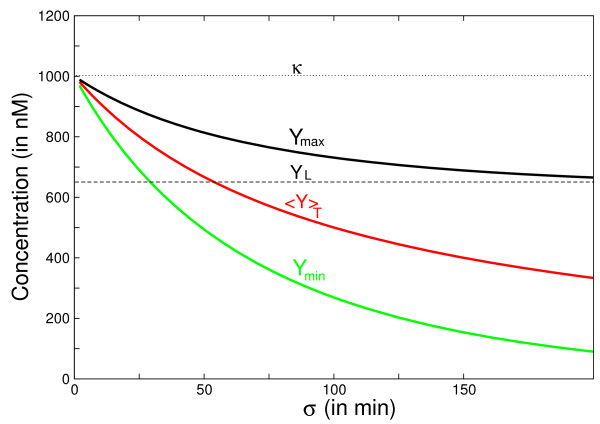
**The graph of extremum values of *Y *in function of *σ *for a simple activation process**. The extremum values of *Y *in function of *σ *for a simple activation process (Fig 1. (a)) are plotted thanks to the equations (2) of the main text. *Y*_*max *_is the maximum reached by the protein, <*Y *>_*T *_is the mean concentration of the protein averaged over one period T, *Y*_*min *_is the minimal value reached by the protein, $\kappa =\frac{\beta }{\alpha }$ (dotted line) represents the stationary state that the protein would attain if the stimulation was constant and *Y*_*L *_(dashed line) is the asymptotic value reached by *Y*_*max *_when the inter-pulse interval *σ *becomes very large. The parameters are: *α *= 0.01 *min*^-1^, *β *= 10 *nM.min*^-1^, *τ *= 100 *min*.

Fig. 3 shows an example of these functions when the inter-pulse *σ *is varied, for a fixed pulse duration *τ*. When *σ *increases from 0 the observables are all decreasing. The maximum *Y*_max _stays within the interval [*Y*_*L*_, *κ*], where *Y*_*L *_is the asymptotic value reached by *Y*_max _(dotted line on Fig. 3, obtained when the denominator of *Y*_max_, eq.(2), equals 1). We note that this level can be controlled by choosing the pulse duration *τ*. This can be useful if *Y *is itself a transcription factor with respect to a target gene *Z *(see next Section).

Another important class of basic activation in molecular biology is the covalent modification of proteins (e.g. the phosphorylation/dephosphorylation), which constitutes the building block of signaling transduction pathways. In some cases, the enzyme promoting the activated form of the protein is submitted to time-periodic variations. Outstanding examples of this situation have been studied in the context of Ca^2+ ^oscillations, whose temporal spiking patterns have been showed to significantly modulate the activity of CaM kinase II [3-6]. The activation/deactivation motif is usually represented as in Fig. 1(b) by drawing two arrows forming a cycle between two molecular states. A third arrow indicates the presence of the external signal *S*(*t*). Here *Y *stands for the concentration of deactivated proteins, and Y* the concentration of activated ones, assuming that the total protein concentration is a conserved quantity, *Y *+ Y* = *Y*_*tot*_. Several theoretical models with different levels of complexity can be used to describe the dynamics of the interconversion between *Y *and Y*. For a recent review, see e.g. [22]. The simplest form of equations which describe the dynamics of the cycle between these proteins is given in the following [23]:

(3) $${\dot{Y}}^{*}=kS(t)(Y_{tot}-Y^{*})-k^{\prime }Y^{*}$$

where *k *and *k' *denote kinetic constants of the respectively activating and deactivating reactions. When *S*(*t*) is a periodic train of square pulses as in the above observables *Y*_max_, *Y*_min _and ⟨*Y*⟩_*T *_can again be analytically calculated as a function of (*τ*, *σ*). However, their expressions are slightly more complicated than those of eqs.(2) due to the difference in time scales for the "on-phase" and "off-phase" of *Y *(*t*). For a fixed value of *τ *the graph of these functions of *σ *is qualitatively similar as seen in Fig. 3. The extrema and the mean values of *Y *(*t*) decrease when the inter-pulse interval *σ *is increased. Let us note that the same behavior is also achieved in the general case of nonlinear equations representing the dynamics of the covalent modifications by mean of the Goldbeter-Koshland model [3,24] (cf. additional file 1).

We conclude from elementary calculations that a pulsatile activation of the simple regulation schemes (1) or (3) provides a way to adjust the variations of *Y *in a range of values which can be controlled by tuning the temporal pattern (*τ*, *σ*) of the pulses.

### Periodic activation of the IFFL

In this Section we show that one basic design which enables one to obtain an optimal output in response to a periodic train of pulses, is the *incoherent feedforward loop *(IFFL) motif studied by Alon and co-workers in the context of transcriptional networks of bacteria [25]. We first give the definition of this motif and then study new properties which appear in this system under periodic stimulations.

#### The type-I Incoherent Feedforward Loop

According to the studies of [18], one of the most recurrent motif arising in a 3-nodes regulatory sub-network is the (type-1) incoherent feedforward loop seen in Fig. 1(c) (the abbreviation is IFFL in the following). Here the transcription factor *X *promotes the expression of both genes *Y *and *Z *but the expression of the latter is repressed by *Y*. In order to simplify the formalism so as to obtain simple analytical estimates, we again consider the logic approximation, where the regulation functions are described by step functions which take only binary values 0 or 1. As in the above, *S*(*t*) = *R*(*X*(*t*)) denotes an input signal emanating from *X *and the dynamics of the system is described by the following equations:

(4) $$\dot{Y}=\beta S(t)-\alpha Y$$

(5) $$\dot{Z}=\beta S(t)H(\theta -Y)-\alpha Z$$

Here the first equation is a simple regulation as introduced in the previous section, and the second equation governs variable *Z *which is activated only when both the signal *S*(*t*) is "on" and when the repressor level *Y *is smaller than the threshold *θ*. We note that for simplicity's sake we have considered same degradation (*α*) and maximal production rates (*β*), for *Y *and *Z*, but this choice is not essential for the results reported below.

The main dynamical property of the IFFL motif which was studied in [25] is its ability to create a pulse in *Z *concentration when it is submitted to a constant stimulus. Once the signal associated to *X *is switched "on", the variable *Z *rises due to its positive regulation by *X*, but this activation is terminated by the repressor *Y *which is also activated by *X*. The duration of the pulse can be quantified by the time *τ*_*θ *_needed by *Y *to reach its repression threshold *θ *after the onset of the signal. For the simple model above, the pulse duration is merely computed as:

(6) $$\tau_{\theta }=-\frac{1}{\alpha }\log(1-\theta /\kappa )$$

This will be called the proper pulse duration of the IFFL system. Thus if the activation lasts longer than the proper pulse *τ*_*θ*_, the system can recover its primary state. In other words the IFFL system can only detect changes of stimulation, meaning that it *adapts *to a constant stimulus. So, as mentioned by Li and Goldbeter [16] in regarding the context of receptor desensitization, in order to reach a given level of synthesis of *Z *in such system, a pulsatile pattern of stimulation must be considered instead of a continuous stimulation. Therefore in the following we consider a periodic stimulus *S*(*t*), which has the form of a square-wave, similar to that studied in the previous Section.

#### Pulsatile periodic activation of the IFFL

**Figure 4:**
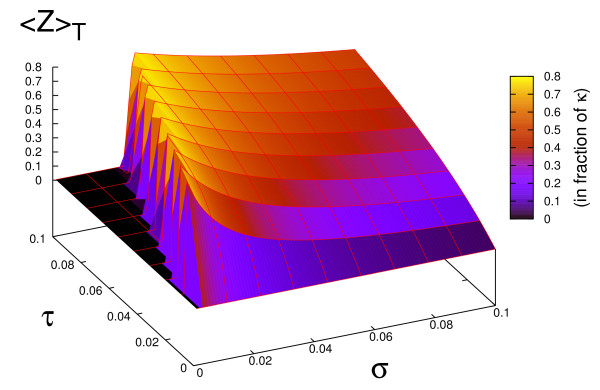
**Response of the IFFL to periodic stimulation in function of *σ *and *τ***. Average response <*Z *>_*T *_is plotted in function of *σ *and *τ *for an I1FFL motif (Fig 1. (c)) stimulated by a pulsatile signal. Parameters *σ *and *τ *are expressed in 1/*α *units and <*Z *>_*T *_in fraction of $\kappa =\frac{\beta }{\alpha }$. Threshold parameter is chosen as *θ *= 0.8 *κ*. This surface plot of <*Z *>_*T *_is analytically computed by using eq.(10) given in the Additional File 1.

**Figure 5:**
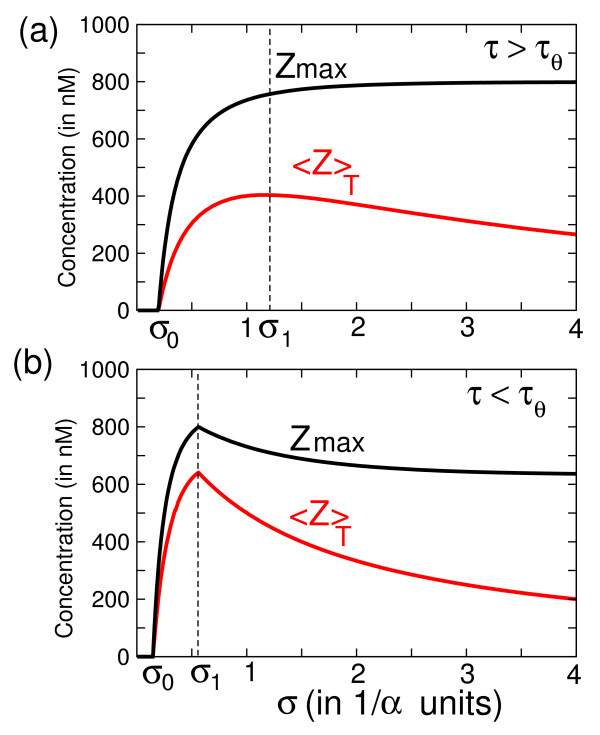
**Response of the IFFL to periodic stimulation in function of *σ***. The average response <*Z *>_*T *_and the maximum of response *Z*_*max *_are represented in function of the inter-pulse interval *σ *with a fixed *τ *for an IFFL motif (Fig 1.(c)) stimulated by a pulsatile signal. *σ *is in 1/*α *units, the concentration is in nM. *σ*_0 _is the minimal inter-pulse interval for the system to respond. The inter-pulse interval *σ*_1 _gives the optimum average response for the system. The numerical simulation was done with the equations (4–5) of the main text and with the following parameters: (a) for the case *τ *> *τ*_*θ*_, *τ *= 2 1/*α *unit. For example, the parameters: *α *= 0.01 *min*^-1^, *β *= 10 *nM.min*^-1^, *θ *= 800 *nM *and *τ *= 200 min give *σ*_0 _~20 min and *σ*_1 _~2 hours. (b) for the case *τ *<*τ*_*θ*_, *τ *= 1 1/*α *unit. For example, the parameters: *α *= 0.01 *min*^-1^, *β *= 10 *nM.min*^-1^, *θ *= 800 *nM *and *τ *= 100 min give *σ*_0 _~15 min and *σ*_1 _~1 hour.

In this Section we show that when the IFFL network is periodically stimulated by a train of pulses, a new property appears regarding the optimization of observables ⟨*Z*⟩_*T *_and *Z*_max_. As illustrated in Fig. 4 and Fig. 5, the average value ⟨*Z*⟩_*T*_, as well as *Z*_max _can reach maximal values for specific choices of the pulse pattern (*τ*, *σ*). We will describe this phenomenon in more detail.

First, the evolution of the repressor *Y *(*t*) is identical to the one obtained in the previous section for a simple regulation, because eq.(4) coincides with eq.(1). However, in what concerns the evolution of the output protein *Z*, a detailed analysis of this system (see additional file 1) shows that two cases should be distinguished according to the size of the pulse duration *τ *compared with the proper pulse *τ*_*θ *_of the IFFL [cf. eq. (6)]. Here we detail only the case of short pulses, corresponding to the condition *τ *<*τ*_*θ*_. Fig. 4 shows a 3D-plot of ⟨*Z*⟩_*T *_computed in function of the pulse parameters (*τ*, *σ*). One observes that the extrema of ⟨*Z*⟩_*T *_form a ridge with a roughly constant slope *τ*/*σ *in the parameter space (*τ*, *σ*). By fixing the pulse duration *τ *Figs. 5 show the existence of maxima in ⟨*Z*⟩_*T*_, as well as in *Z*_max _in Fig. 5b, reached for a critical inter-pulse interval *σ*_1_. Another feature of these observables shown in Figs. 5 is that they start to increase from zero only if the inter-pulse interval *σ *is above a minimum value *σ*_0_. When *τ *<*τ*_*θ*_, the values of *σ*_0 _and of *σ*_1 _can be analytically computed as follows (cf. Suppl. Info.):

(7) $$\sigma_{0}=\frac{1}{\alpha }\log\left(1+\frac{\kappa }{\theta }(e^{\alpha \tau }-1)\right)-\tau$$

(8) $$\sigma_{1}=-\frac{1}{\alpha }\log\left(1-\frac{\kappa }{\theta }(1-e^{-\alpha \tau })\right)-\tau$$

The latter expression can serve to estimate the optimal interspike interval *σ*_1 _which should be waited for between the pulses to maximize the response of the IFFL network. We note that when *ατ *is small, one can use the first-order approximation *σ*_0 _~*σ*_1 _~*τ *(*κ*/*θ*-1), where *κ *is the equilibrium concentration of *Y *proteins, and *θ *<*κ *is the repression threshold of *Y *with respect to gene *Z*. Our estimate means that provided *ατ *is small enough, the optimal pulse pattern is given by only one condition on the ratio between the on/off phases, i.e. *σ*_1_/*τ *~*κ*/*θ *- 1. It does not impose a unique value for the pulse duration *τ*. Furthermore, it can be seen that the height of the optimal ⟨*Z*⟩_*T *_and *Z*_max _is of the order of *θ*. Thus the average and maximum productions of *Z *are optimized when *θ *is close to its maximal possible value, which is bounded by *κ*. But then *σ*_1 _≪ *τ*, meaning that in this case the optimal rest interval would be smaller than the pulse duration. Therefore for the IFFL with a high repressor threshold, optimal trains of square pulses would seem to be alike in a constant stimulation. However, for this system, there is a crucial difference between the situation of constant stimulus, where *Z *falls to zero, because it is completely repressed by *Y*, and the other situation where the activation of *Y *is periodically interrupted during for a small lapse of time. In real biochemical systems, however, the value of the threshold *θ *cannot be arbitrarily chosen, and the implementation of our proposed principle depends on the context, being for instance the one of genetic regulation or the one of intracellular signaling. Based on plausible parameter values found in the literature, Table 1 gives examples of optimal pulse patterns predicted by our simple model in the case of genetic expression, eqs.(4)-(5), or in the case of signaling cycles, eqs.(3)–(5). The first line gives optimal interpulse durations *σ*_1 _for a simple IFFL composed of three genes with parameters in the range given in the experimental work of [26]. The second line gives an estimation of *σ*_1 _for an IFFL whose node *Y *is a signaling cycle. The numerical values for the parameters of the signaling cycle, (*k' *= *k *= 1 s^-1^, *Y*_*tot *_= 2 *μ*M), are based on values estimated in [27].

**Table 1:** Examples of plausible numerical values for the different parameters

Simple IFFL in bacteria [26]	*α *= 0.01*min*^-1^	*β *= 100 nM.*min*^-1^	*θ *= 500 nM	1 to 10 min	25 to 390 min
IFFL with a signaling cycle [27]	*K' *= 1 *s*^-1^	*k *= 1 s^-1^	*θ *= 500 nM	1 s	2 s

In conclusion one sees that the IFFL network motif (Fig. 1(c)) studied by Alon *et al*. has a nice ability to provide one of the simplest mechanisms which maximizes the average synthetized protein ⟨*Z *⟩_*T *_when periodically stimulated with a train of pulses.

### Mixed regulatory networks with a "Diamond" IFFL

The periodic stimulation of the IFFL network with train of pulses reveals an interesting property of optimal response with respect to the pulse pattern. In this section we slightly extend the IFFL motif to obtain a design which improves its ability of bandpass filtering, i.e. the property to induce a non zero response only for a finite range of pulse periods. We consider the "diamond IFFL" motif depicted in Fig. 1(d). The scheme is analog to the IFFL but the activation of the target gene takes two nodes instead of only one. In fact, this extension of the IFFL can be studied in the gene network seen in Fig. 1(d). It can also be implemented in a mixed regulatory network which blends signaling and gene nodes in the same interaction graph (cf. Fig. 1(e)). This graph may schematically represent a transduction network consisting of a crosstalk between two pathways converging with opposite interactions in the same genetic system. A similar scheme has been used in the study of the formation of long term memory at the molecular level [15]. The comparison between the models of the literature and the outcome of our analysis will be provided in the Discussion section.

We again use the simplified description of eq.(3) for the dynamics of the covalent modification cycles, with variable $Y_{1}^{*}$ and $Y_{2}^{*}$, and the logic regulatory functions for the activation of the promoter of *Z*. The equations of the system depicted on Fig. 1(e) can be written as previously with the following dynamics for the proteins $Y_{1}^{*}$, $Y_{2}^{*}$ and *Z*:

(9) $${\dot{Y}}_{1}^{*}=kS(t)(Y_{1tot}-Y_{1}^{*})-k^{\prime }Y_{1}^{*}$$

(10) $${\dot{Y}}_{2}^{*}=kS(t)(Y_{2tot}-Y_{2}^{*})-k^{\prime }Y_{2}^{*}$$

(11) $$\dot{Z}=\beta H(Y_{1}^{*}-\theta_{1})H(\theta_{2}-Y_{2}^{*})-\alpha Z$$

In this simple setting where identical kinetic parameters are assumed for the covalent modification cycles 1 and 2, the time-evolutions of $Y_{1}^{*}$ and $Y_{2}^{*}$ are synchronized by the periodic signal *S*(*t*). In this case, eq. (11) shows that the expression of *Z *gene is activated only when *θ*_1 _<$Y_{1}^{*}$ <*θ*_2 _(*i *= 1, 2), which entails that the relation *θ*_1 _<*θ*_2 _must be assumed in order to have a non zero production of *Z*. This assumption means that the affinity of the activator for binding the promoter of *Z *gene is higher than the affinity of the repressor for the same promoter.

**Figure 6:**
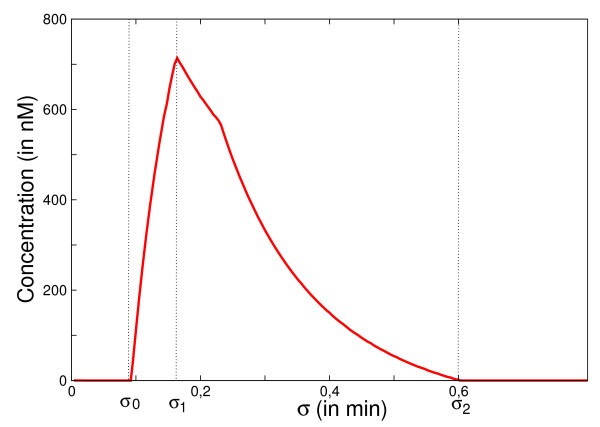
**Response of the Diamond IFFL motif with signaling cycles to periodic stimulation in function of *σ***. The average response <*Z *>_*T *_of the protein Z of the Diamond IFFL motif with signaling cycles (Fig. 1 (e)) is shown for a pulsatile stimulation. *σ*_0 _is the minimal inter-pulse interval for the system to respond. The inter-pulse interval *σ*_1 _gives the optimum average response for the system and *σ*_2 _is the maximal inter-pulse interval for the system to respond. The numerical simulation was done with the equations (9–11) and with the following parameters: *k *= 4 *min*^-1^, *k' *= 2 *min*^-1^, *Y*_1*Tot *_= *Y*_2*Tot*_= 500 nM, *α *= 0.01 *min*^-1^, *β *= 10*nM.min*^-1^, *θ*_1 _= 180 *nM*, *θ*_2 _= 230 *nM*, *τ *= 0.1 *min*.

We consider periodic stimulations of this system with a pulse pattern (*τ*, *σ*) such that *σ *is varying and the pulse duration *τ *<$\tau_{\theta_{1}}$ is fixed (cf. eq.(6)). Fig. 6 shows the variation of the mean value of synthesized *Z*, denoted by ⟨*Z*⟩_*T*_. As for the IFFL, there is a lower limit of the inter-spike interval *σ*_0 _in which no production of the target gene *Z *occurs. But once *σ *is increased above *σ*_0 _there is a fast growth of ⟨*Z*⟩_*T *_which culminates at *σ*_1 _which can be computed in the same way as before (eq.(8)). If *σ *is further augmented, ⟨*Z*⟩_*T *_registers a decrease which reaches 0 when *σ *> *σ*_2 _on the graph. This property is due to the existence of a new node (activator Y1) in the "diamond" IFFL, which ceases to activate the target gene Z once *Y*_1 _max becomes smaller than its activation threshold of Z. Thus the new feature of the pulse-response of ⟨*Z*⟩_*T *_is that (for a fixed *τ*) one obtains only a finite interval [*σ*_0_, *σ*_2_] of inter-pulse intervals in which the protein *Z *is produced. Therefore, the new property appearing in this system, as compared with the standard IFFL, gives us the possibility to use this architecture as a bandpass filtering, allowing the gene to respond only to specific periodic trains of pulses, with the inter-pulse *σ *belonging to a limited range [*σ*_0_, *σ*_2_].

### Optimal output in response to bursting oscillations

The network motifs studied in the previous Sections can be combined in modules that can be linked in a larger network to process complex signals. We show in this Section that by linking two diamond IFFL motifs one obtains a class of regulatory networks which are able to selectively respond to bursting oscillations.

Bursting oscillations are time-dependent signals typically emitted by neuron cells [28]. Their temporal pattern is formed by trains of spikes alternating with refractory periods. The neuronal signal is a depolarization wave created by the dynamics of ionic channels in the neuron membranes. However, in the vicinity of a synapse, this electrical signal transforms into a chemical signal affecting the receptors of the post-synaptic neuron. It thereby propagates its influence in the molecular regulatory networks of this neuron. Consequently, a bursting neuronal signal can potentiality impact on the gene expression of the post-synaptic neuron. Therefore it is worthwhile to identify simple topologies of regulatory networks with the ability to be sensitive and selective to bursting oscillations.

In order to explore this question we consider the network represented in Fig. 1(f), which combines in series two diamond IFFL motifs. Each node of Fig. 1(f) is represented by single species but in practice the proteins *Y*_1 _and *Y*_2 _on this graph could be replaced by covalent modification cycles as the ones depicted in Fig. 1(e). In this case Fig. 1(f) corresponds to a regulatory system where an input signal influences the expression of a target gene *W *by the intermediary of a signaling pathway, in which 2 phosphorylation/dephosphorylation cycles respectively activate and repress the promoter of the intermediate gene *Z*. The corresponding protein activates in turn the repressor *V*_1 _and the activator *V*_2 _of a target gene *W*.

Employing similar notations as in the previous Sections, the dynamics of the network on Fig. 1(f) can be described by the following system:

(12) $${\dot{Y}}_{1}=\beta_{1}S(t)-\alpha_{1}Y_{1}$$

(13) $${\dot{Y}}_{2}=\beta_{1}S(t)-\alpha_{1}Y_{2}$$

(14) $$\dot{Z}=\beta_{1}H(Y_{1}-\theta_{1})H(\theta_{2}-Y_{2})-\alpha_{1}Z$$

(15) $${\dot{V}}_{1}=\beta_{2}H(Z-\theta_{3})-\alpha_{2}V_{1}$$

(16) $${\dot{V}}_{2}=\beta_{2}H(Z-\theta_{4})-\alpha_{2}V_{2}$$

(17) $$\dot{W}=\beta_{2}H(V_{1}-\theta_{5})H(\theta_{6}-V_{2})-\alpha_{2}W$$

**Figure 7:**
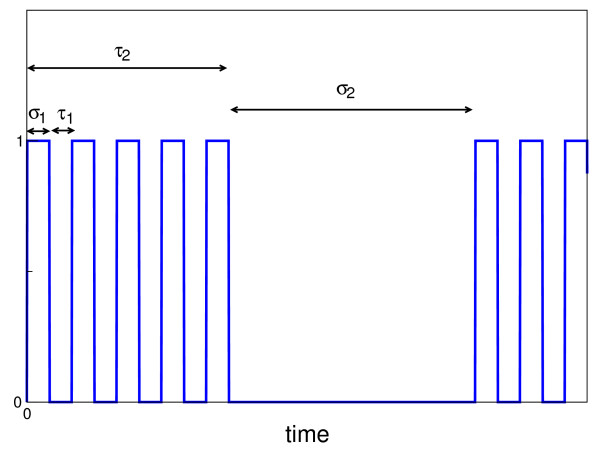
**Bursting signal**. The signal is composed of 2 signals of different time scale. The first is a rapid pulsatile signal with the duration of the "on-phase" denoted by *τ*_1 _and the silent phase between the pulses denoted by *σ*_1_. The second signal is two orders of magnitude slower. The duration of the "on-phase" is denoted by *τ*_2 _and the "off" phase between the pulses, is denoted by *σ*_2_. For practical reasons, the graph is not at real scale.

To model the input bursting signal *S*(*t*) we consider a time-periodic pattern characterized by four time intervals (*τ*_1_, *σ*_1_, *τ*_2_, *σ*_2_) (cf. Fig. 7). The period of the signal is given by *T *= *τ*_2 _+ *σ*_2_, where *τ*_2 _is the duration of the bursting phase and *σ*_2 _is the quiescent period. The bursting phase is defined by a series of square spikes in which *τ*_1 _is the width of the spike and *σ*_1 _is the interspike interval. The quiescent period which separates the bursting phases is given by *σ*_2_. Thus the signal is characterized by two different time-scales *τ*_1 _+ *σ*_1 _≪ *τ*_2 _+ *σ*_2_. In the numerical simulations reported below these time scales are separated by two orders of magnitude, reflecting the typical temporal differences between the dynamics in signaling cascades and in genetic regulations (cf. Table 1).

**Figure 8:**
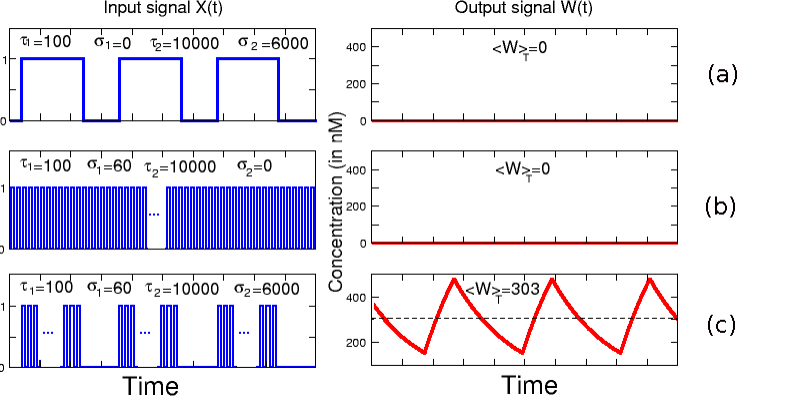
**Response of the double DIFFL to a bursting signal**. The motif of double DIFFL (Fig. 1(f)) is stimulated with 3 different periodic input signals *X*(*t*) (on the left). The 3 graphs on the right give the response of the output *W *(*t*). The numerical simulations were done with the following parameters *τ*_1 _= 100 s and *τ*_2 _= 10000 s are fixed. The other parameters are: *α*_1 _= 0.01 *sec*^-1^, *β*_1 _= 10 *nM.sec*^-1^, *α*_2 _= 0, 006 *min*^-1 ^*β*_2 _= 6 *min*^-1^, *θ*_1 _= *θ*_5 _= 700 *nM*, *θ*_2 _= *θ*_6 _= 800 *nM*, *θ*_3 _= *θ*_4 _= 50 *nM*. (a) with *σ*_1 _= 0 s, *σ*_2 _= 100 *min *(b) with *σ*_1 _= 60 s, *σ*_2 _= 0 *min *(c) *σ*_1 _= 60 s, *σ*_2 _= 100 *min*. For practical reasons, the representations of the pulsatile signals are not at real scale.

When the network represented in Fig. 1(f) is stimulated by the signal *S*(*t*) described above, it has the property to selectively recognize temporal patterns of bursting oscillations, allowing the target gene to be maximally transcribed in some conditions. To illustrate this point Fig. 8 shows the time-evolution of *W *in response to various periodic stimulations *S*(*t*). When the system is submitted to periodic trains of square waves without bursting (Fig. 8(a), *σ*_1 _= 0), the gene *W *is not expressed. Likewise if the stimulation consists of a long train of spikes without any quiescent period (Fig. 8(b)), the average level of *W *remains negligible. However, if we stimulate the motif with a specific bursting signal (Fig. 8(c)), the system gives a non-zero response. More generally, the striking feature of the network of Fig. 1(f) is to exhibit a non vanishing response only in a given range of pulse patterns. Moreover, if the time intervals *τ*_1 _and *τ*_2 _are fixed, the system possesses a set of maxima for some optimal values of (*σ*_1_, *σ*_2_). In view of of Fig. 8(a–c), the system behaves as it filtered out low as well as high frequencies. But this conclusion is misleading since when high and low frequencies are mixed in the same input signal in the form of bursting oscillations, the system displays a non zero response in the evolution of *W*, with the possibility of optimizing the average level of *W *over one period.

The reason why the network in Fig. 1(f) manifests a large response to bursting oscillations is a direct consequence of the results discussed in the previous Sections. Let us again suppose that the active phase of the bursting lasts *τ*_2 _and the quiescent interval is *σ*_2_. During this active phase *τ*_2_, and assuming the spike duration *τ*_1 _is fixed, an optimal output in the level of the intermediate protein *Z *is produced if the interspike interval *σ*_1 _is chosen as the one which maximizes the response curve of Fig. 6. For example, using the parameter of that figure, this corresponds to *σ*_1 _≃ 1/(4*α*_1_). On the other hand, during the refractory period *σ*_2 _of the bursting oscillations, the activity of *Z *returns to zero. Thus the time-evolution of the intermediate protein *Z *is close to a periodic train of square pulses with time pattern (*τ*_2_, *σ*_2_) characterizing respectively its "on" and "off" phases. Now this variable feeds the regulation functions of the second subnetwork *V*_1 _- *V*_2 _- *W*. But for a fixed *τ*_2 _this subnetwork responds in an optimal way if the quiescent phase is chosen again as the critical *σ*_2 _which maximizes a response curve like the one shown in Fig. 6. For example, using the same parameter as for this figure, *σ*_2 _would correspond to 1/(4*α*_2_). Therefore, since the signaling time scale 1/*α*_1 _is much smaller than the one of gene regulation 1/*α*_2_, we conclude that the temporal pattern which maximizes the response in *W *is *σ*_1 _≪ *σ*_2_. This agrees with the concept of bursting oscillations.

### The interlocked negative feedback loop

The network motifs analyzed in the previous Sections can give rise to an optimum period of stimulation corresponding to a maximum in the *average *concentration of the output proteins. It is also interesting from a physiological point of view, to find biological networks that give an optimum frequency maximizing the oscillatory *amplitude *of the output. So, we consider another basic network motif which provides a maximal response in function of a periodic stimulation. The motif network, which is shown in Fig. 1(g), is similar to the IFFL, but the repressor *Y *in addition to being activated by X is activated by Z in a feedback loop. Furthermore, Z is not directly activated by X. This negative loop is not a gene regulatory motif in the sense defined by Alon et al., but we still call it a motif as it is the building block of several molecular biology oscillators [29]. We note that often this regulatory motif is not purely genetic, but the negative control is induced by a post-transcriptional regulation [30]. Furthermore, when the species *Z *not only activates *Y *but also exerts an auto-activation, the system can become a biological oscillator. In fact this positive auto-regulation is essential in order to display self-sustained oscillations. For example, minimal models of circadian oscillators have been proposed, involving the pattern of activation seen in Fig. 1(g)[31]. Likewise, minimal models for the cellular cycle have also been proposed in this way [32]. This network motif also supports a model for the oscillatory dynamics of *p*53 [33], oscillations in Neurospora [34]. It has also been studied in a synthetic biology perspective [35]. Other examples exist (cf. Table 2), like for instance a model given by Song et al. [36] who also use the interlocked negative feedback loop to describe a model of memory formation.

**Table 2:** Different models of the interlocked negative feedback loop. This table shows various regulators which are involved in an interlocked negative feedback loop.

Model	Cellular cycle [32]	P53 [33]	Synthetic system [35]	Circadian rhythms (Neurospora) [34]
Y (Repressor)	CLB	Mdm2	LacI	FRQ
Z (Activator)	CLN	P53	NRI	WC-1, WC-2

All these examples have been proposed as models of biological clocks, because they admit autonomous oscillations of the produced protein concentrations. In absence of the positive auto-regulation of the activator, however, it can be shown that no autonomous oscillations are possible. This is the case we consider for the network motif depicted in Fig. 1(g). But even in this simpler network, interesting oscillatory behaviors can appear if the variable *Z *(or *Y*) is influenced by periodic variations coming from *X*. This periodic activation can be caused by a signal associated with an autonomous oscillator, or by the outcome of a signaling pathway which is periodically stimulated, as considered in the previous Section. In all the cases we represent the periodic activation of unit *X *in Fig. 1(g) by a signal *S*(*t*), and the dynamics of the protein concentrations *Y *and *Z *are determined by the following equations:

(18) $$\begin{array}{c} \dot{Y}=(\beta_{0}+\beta_{1}S(t))R^{+}(Z)-\alpha Y\\ \dot{Z}=\beta_{2}R^{-}(Y)-\alpha Z \end{array}$$

In this system, the signal *S*(*t*) associated with *X*(*t*) can be a periodic square pulse of amplitude 1, *R*^+^(*Z*) is an activating regulation function, like the Hill function introduced above, and *R*^-^(*Y*) = 1 - *R*^+^(*Y*) is a repressing function.

The resonance response is much clearer in this case if the input signal is sinusoidal rather than a square signal. The work performed by Lipan and Wong [37] proposes the use of oscillatory signals for studying genetic networks. These authors have devised a promising experimental procedure which consists of activating and deactivating the promoter efficiency with the use of electromagnetic fields. The net effect of their procedure amounts to periodically modulating the expression rate of genes.

**Figure 9:**
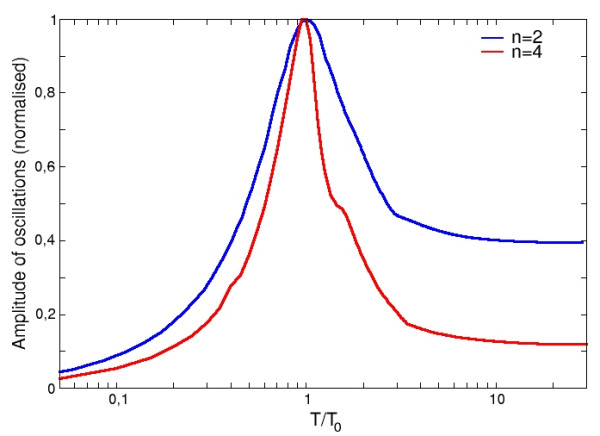
**Response of the interlocked negative feedback loop to a harmonic stimulation**. The response in *amplitude *of the interlocked negative feedback loop (Fig 1.(g)) to an harmonic stimulation in function of the period of stimulation T is shown. The numerical simulation was done with the equations (18) and with the following parameters: *β*_0 _= 150 *nM.min*^-1^, *β*_1 _= 100 *nM.min*^-1^, *β*_2 _= 100 *nM.min*^-1^, *θ*_1 _= 50 *nM*, *θ*_2 _= 50 *nM*, *α *= 0.01 *min*^-1^, n = 2 or 4. With these parameters, we have *T*_0 _~ 6 hours.

When the periodic activation is absent, *β*_1 _= 0, the system possesses a non-zero steady state (*Y*_*s*_, *Z*_*s*_) which can be shown to always be a stable focus. The corresponding frequency *ω*_0 _is easily determined by computing the imaginary part of the Jacobian eigenvalues. Thus, if the system is drawn away from equilibrium, it will return to its steady state by exhibiting transient oscillations with the frequency *ω*_0_. This frequency can also be revealed by periodically modulating the expression rate of *Y*. In this case we suppose that the modulation is operated with a frequency varying in a range around *ω*_0_. Numerical simulations of eqs. (18) show that in this range the network dynamics give rise to a resonance which coincides with *ω*_0_. In Fig. 9, we can see a maximum in the amplitude response of protein Y. A typical order or magnitude for the resonant period obtained from plausible parameters is *T*_0 _~300 min. Moreover we observe that the resonance width mainly depends on the stiffness of the regulatory functions, quantified here by the cooperativity coefficient *n *of the Hill function. To explain this property, an easy but instructive example which can be analytically dealt with, considers identical thresholds *θ *for the regulatory Hill functions *R*^+^(*Z*) and *R*^-^(*Y*), and also assume *β*_0 _= *β*. The steady state for *Y *and *Z *is symmetric, i.e. *Y*_*s *_= *Z*_*s *_= *κ*/2 (we recall that *κ *= *β*/*α*). The natural frequency can be written as *ω*_0 _= *nα*/2, where *n *is the Hill coefficient of functions *R*^+^(*Z*) and *R*^-^(*Y*). Since, in absence of periodic stimulations, the damping of the oscillations is characterized by *α*, we can estimate the "quality" factor of this resonance with the ratio *ω*_0_/*α *= *n*/2. This indeed depends only on the Hill coefficient *n*. Examples of resonances with *n *= 2 and *n *= 4 are illustrated in Fig. 9.

## Discussion

In this study we have looked at simple molecular networks showing the ability to maximize their responses to periodic stimulations. Using analytical and numerical tools, we have identified two types of basic network motifs, and some of their straightforward extensions, which possess this property (Fig. 1). The first of thes motifs is the Incoherent Feedforward Loop network (IFFL), for which we have shown that periodic activations with a train of square pulses lead to maximizing the time-average production of the output protein over one period provided that the pulse and the inter-pulse intervals are adequately chosen (Fig. 4). When the pulse duration is short enough, this optimization also applies to the maximal concentration of the output protein reached over one period (Fig. 5b). The mathematical analysis of this optimization property is simple, although it does not reckon as do most current studies which focus on this topic, with the assumption of small amplitude stimulations, which allows to work in the framework of linear filter analysis. We have also pointed out another network motif, namely the interlocked negative feedback loop which has the property to exhibit a maximum in the amplitude of oscillations when it is submitted to periodic stimulations. In this case the maximization is observed in the linear regime (i.e. for small amplitude of the stimulations) and it can be described as a standard phenomenon of resonance.

The interlocked negative loop is a well-studied motif in the context of oscillating regulatory networks (cf. examples given on Table 2). In all these examples the auto-activation of one of the two nodes of the network is imperative in order to produce sustained oscillations. Here we pinpoint a property of the interlocked feedback loop that exists without or with only a weak auto-activation. In this case, the system relaxes towards a stationary state in absence of periodic stimuli. It nevertheless possesses the ability to show up a resonant behavior which can be waked by periodic stimulations. This phenomenon might be important in some situations. For example the protein P53 is engaged in many regulation processes and it is known to oscillate with a period of 5.5 hours in some conditions. In [38] the authors show that the amplitude of oscillations of P53 is variable whereas the period of oscillations has a spectacular regularity. We speculate that even in absence of autonomous oscillations this system might show up an amplified response to external periodic stimulations (e.g. a periodic exposure to UV rays) because of the resonance phenomenon. A similar remark can be made regarding regulatory systems related to stress responses as in the SOS regulator LexA of *E. coli *or in the NF-*κ*B system.

As an illustrative application of the periodic activation of the diamond IFFL motif we believe that the feature studied in the present paper reveals the principle behind an intriguing phenomenon studied by Smolen et al. [15]. In this reference, the authors proposed a model for the formation of long term memory (LTM), a process which is based on the LTP phenomenon (Long Term Potentiation) in neurons and on the subsequent strengthening of synapses. The LTP appears in a synapse when the postsynaptic cell is submitted to some external signal, for instance in the form of glutamate pulses of significant amplitude. Then this signal induces the phosphorylation of transcription factors which genetically activate the production of new proteins in order to strengthen connections between synapses or to create new synapses. On the other hand, it has been shown that in order to learn, several animals or organisms need to receive stimulations in repeated sessions (a well-studied example is the aplysia, see [39] for a review). Interestingly these sessions must be spaced with a minimal interval. Massed training produced significantly shorter lasting memory as experimentally shown in [40]. Thus the genetic network underlying this regulation must respond only if the learning sessions are sufficiently spaced.

The model of Smolen et al. (Fig. 5 of [15]) had the same structure as the diamond IFFL shown in Fig. 1(e). It postulated the existence of two transcriptional factors, respectively an activator and a repressor, whose phosphorylated forms are activated by an external signal, allowing them to act on the promoter of a gene involved in the formation of LTM (e.g. the *creb *genes). The purpose of this modeling was to show the marked differences in the transcription rate of the LTM genes between the case of "massed stimuli" and the case of "spaced stimuli", as was experimentally observed e.g. by Yin et al. [2]. In fact an outstanding property of their model was the existence of a maximum in the transcription rate of the LTM genes, in function of the interspike interval (here corresponding to the rest time between the learning sessions). However the definite mechanism underlying this property is not easy to identify in that system, as the model counted a half dozen of nonlinear equations, with nearly 20 parameters to be chosen. Despite this complexity the authors did conclude by suggesting that a general mechanism enabling the tuning of the response of a gene promoter to an optimal stimulus frequency could be the presence of two competing processes with different sensitivities acting on this gene. In this article we have achieved a minimal model which puts this intuition on firm grounds. Our analysis shows that the presence of two competing processes related by the IFFL mechanism is indeed a key ingredient, a higher affinity of the activator compared with the one of the repressor for the target gene promoter is another one.

The results of Li and Goldbeter [16] mentioned in the introduction are another rare example found in the literature where a maximization process of the response in a molecular network is shown to critically depend on some temporal patterns of activation. Although this study is based on analytical calculations the basic ingredient which explains the observed property is not transparent. Although the link of this model with the IFFL motif is not established we are currently working on a generalization of the optimization principle identified in the IFFL responses in order to place the two studies in the same framework.

Based on typical parameter values met in different systems (genetic, signaling), Table 1 provides some estimates of the pulse and of the inter-pulse intervals which allow for maximization of the average production of target proteins. Since these estimates seem compatible with plausible experimental values, they prompt experimental implementations of the scheme. For example, several IFFL regulatory motifs have been recognized in a genome map of *E. coli *[18]). It would be interesting to stimulate one of these loops with trains of periodic pulses in order to discover whether the predicted maximization is feasible in practice. Utilization of thermosensitive promotors may be one possibility for the implementation of the periodical stimulations [41]. Nevertheless we note that the phenomena presented here may be sensitive to molecular noise and to environmental perturbations. Presumably, the envisaged experiments might be less difficult to achieve in eukaryote organisms where the genetic regulations involve more proteins than in prokaryotes, in this way, molecular noise could be reduced.

A general issue concerning the concept of network motifs, like the IFFL, is that such a module is in fact embedded in larger and more complex regulatory networks [42]. This weakens the actual meaning of modular subnetworks. In this regard, however, by considering time dependent signals we propose that frequency selectivity mechanisms like the ones studied here, can give more sense to the modular approach. Finally we note that from a biomedical perspective the question of optimal response in molecular networks could be especially relevant in situations where a maximal protein synthesis would be beneficial in periodic stimulation-based therapies. For example some innovative treatments of neural based diseases like Parkinson's, or of severe depressions, use a novel technique consisting of deep brain stimulations [43]. The patient wears a pacemaker linked to electrodes implanted in some brain nuclei. Electrical stimulations are then transmitted at a given frequency. Although this type of clinical treatment has been reported to lead to spectacular relief, the question of fine tuning the frequency of the pacemaker to achieve an optimal result is empirically solved for the time being [44]. A better understanding of the time-dependent responses of intracellular signaling pathways could be very relevant in these situations. In a different context, the traditional periodic intake of medicines is another example where optimization will emerge from rational thinking in terms of optimal pulsatile stimulations.

## Conclusion

The mechanisms presented in this article identify new possible strategies which can be employed by a cell to integrate time-dependent information provided by the environment. The studied network motifs offer the attractive possibility of selecting a signal according to its temporal structure or to its frequency content. Only messages which for instance, which have a minimal inter-pulse duration are allowed to pass. The identified mechanisms are simple and based on known network motifs in the literature, they can be embodied in existing organisms. They could also be implemented by means of synthetic biology [41,45].

## Methods

### Numerical simulations

Numerical simulations were made with programs written in C language and the Runge-Kutta routines from the GNU Scientific Library. The simulation codes of the models which were used to obtain the numerical results reported in the paper (e.g. Figs. 5, 6, 8 and 9) are provided in the additional file 2 (with the name "archive-codes").

## Authors' contributions

Conceived and designed the experiments: AC, JAS. Analyzed the data: AC, J-AS. Wrote the paper: AC, JAS. All authors read and approved the final manuscript.

## Supplementary Material

Additional File 1 Supplementary Information. Additional details are given about the mathematical analysis of the periodic activation of the IFFL network by a train of square pulses and about a comparison of our study with the frequency coding analysis performed by Goldbeter *et al*. in [3]. Click here for file

Additional File 2 archive-codes. Computer codes in C language used for the numerical simulations. Click here for file

## References

[B1] Goldbeter A (1996). Biochemical Oscillations and Cellular Rhythms: The molecular bases of periodic and chaotic behaviour.

[B2] Yin JC, Vecchio MD, Zhou H, Tully T (1995). CREB as a memory modulator: induced expression of a dCREB2 activator isoform enhances long-term memory in Drosophila. Cell.

[B3] Goldbeter A, Dupont G, Berridge MJ (1990). Minimal model for signal-induced Ca2+ oscillations and for their frequency encoding through protein phosphorylation. Proc Natl Acad Sci USA.

[B4] Dupont G, Goldbeter A (1992). Protein phosphorylation driven by intracellular calcium oscillations: a kinetic analysis. Biophys Chem.

[B5] Koninck PD, Schulman H (1998). Sensitivity of CaM kinase II to the frequency of Ca2+ oscillations. Science.

[B6] Dupont G, Houart G, Koninck PD (2003). Sensitivity of CaM kinase II to the frequency of Ca2+ oscillations: a simple model. Cell Calcium.

[B7] Mettetal JT, Muzzey D, Gómez-Uribe C, van Oudenaarden A (2008). The frequency dependence of osmo-adaptation in Saccharomyces cerevisiae. Science.

[B8] Dolmetsch RE, Xu K, Lewis RS (1998). Calcium oscillations increase the efficiency and specificity of gene expression. Nature.

[B9] Hersen P, McClean MN, Mahadevan L, Ramanathan S (2008). Signal processing by the HOG MAP kinase pathway. Proc Natl Acad Sci USA.

[B10] Gomez-Uribe C, Verghese GC, Mirny LA (2007). Operating Regimes of Signaling Cycles: Statics, Dynamics, and Noise Filtering. PLoS Comput Biol.

[B11] Cox CD, McCollum JM, Austin DW, Allen MS, Dar RD, Simpson ML (2006). Frequency domain analysis of noise in simple gene circuits. Chaos.

[B12] Shankaran H, Wiley HS, Resat H (2007). Receptor downregulation and desensitization enhance the information processing ability of signalling receptors. BMC Syst Biol.

[B13] Shankaran H, Resat H, Wiley HS (2007). Cell surface receptors for signal transduction and ligand transport: a design principles study. PLoS Comput Biol.

[B14] Locasale JW (2008). Signal duration and the time scale dependence of signal integration in biochemical pathways. BMC Syst Biol.

[B15] Smolen P, Baxter DA, Byrne JH (1998). Frequency selectivity, multistability, and oscillations emerge from models of genetic regulatory systems. Am J Physiol.

[B16] Li Y, Goldbeter A (1989). Frequency specificity in intercellular communication. Influence of patterns of periodic signaling on target cell responsiveness. Biophys J.

[B17] Segel LA, Goldbeter A, Devreotes PN, Knox BE (1986). A mechanism for exact sensory adaptation based on receptor modification. J Theor Biol.

[B18] Alon U (2006). An Introduction to Systems Biology.

[B19] Wolf DM, Arkin AP (2003). Motifs, modules and games in bacteria. Curr Opin Microbiol.

[B20] Gouzé J, Sari T (2002). A class of piecewise linear differential equations arising in biological models. Dyn Syst.

[B21] Glass L, Kauffman S (1973). The logical analysis of continuous, non-linear biochemical control networks. J Theor Bio.

[B22] Ventura AC, Sepulchre JA, Merajver SD (2008). A hidden feedback in signaling cascades is revealed. PLoS Comput Biol.

[B23] Heinrich R, Neel BG, Rapoport TA (2002). Mathematical models of protein kinase signal transduction. Mol Cell.

[B24] Goldbeter A, Koshland DE (1981). An amplified sensitivity arising from covalent modification in biological systems. Proc Natl Acad Sci USA.

[B25] Mangan S, Itzkovitz S, Zaslaver A, Alon U (2006). The incoherent feed-forward loop accelerates the response-time of the gal system of Escherichia coli. J Mol Biol.

[B26] Rosenfeld N, Young JW, Alon U, Swain PS, Elowitz MB (2005). Gene regulation at the single-cell level. Science.

[B27] Kholodenko BN (2000). Negative feedback and ultrasensitivity can bring about oscillations in the mitogen-activated protein kinase cascades. Eur J Biochem.

[B28] Izhikevich EM (2006). Bursting. Scholarpedia.

[B29] Barkai N, Leibler S (2000). Circadian clocks limited by noise. Nature.

[B30] Guantes R, Poyatos JF (2006). Dynamical principles of two-component genetic oscillators. PLoS Comput Biol.

[B31] Gonze D, Halloy J, Goldbeter A (2002). Robustness of circadian rhythms with respect to molecular noise. Proc Natl Acad Sci USA.

[B32] Tyson JJ, Csikasz-Nagy A, Novak B (2002). The dynamics of cell cycle regulation. Bioessays.

[B33] Ciliberto A, Novak B, Tyson JJ (2005). Steady states and oscillations in the p53/Mdm2 network. Cell Cycle.

[B34] Dunlap JC, Loros JJ (2004). The neurospora circadian system. J Biol Rhythms.

[B35] Atkinson MR, Savageau MA, Myers JT, Ninfa AJ (2003). Development of genetic circuitry exhibiting toggle switch or oscillatory behavior in Escherichia coli. Cell.

[B36] Song H, Smolen P, Av-Ron E, Baxter DA, Byrne JH (2007). Dynamics of a minimal model of interlocked positive and negative feedback loops of transcriptional regulation by cAMP-response element binding proteins. Biophys J.

[B37] Lipan O, Wong WH (2005). The use of oscillatory signals in the study of genetic networks. Proc Natl Acad Sci USA.

[B38] Geva-Zatorsky N, Rosenfeld N, Itzkovitz S, Milo R, Sigal A, Dekel E, Yarnitzky T, Liron Y, Polak P, Lahav G, Alon U (2006). Oscillations and variability in the p53 system. Mol Syst Biol.

[B39] Kandel ER (2004). The molecular biology of memory storage: a dialog between genes and synapses. Biosci Rep.

[B40] Carew TJ, Pinsker HM, Kandel ER (1972). Long-Term Habituation of a Defensive Withdrawal Reflex in Aplysia. Science.

[B41] Gardner TS, Cantor CR, Collins JJ (2000). Construction of a genetic toggle switch in Escherichia coli. Nature.

[B42] Mazurie A, Bottani S, Vergassola M (2005). An evolutionary and functional assessment of regulatory network motifs. Genome Biol.

[B43] Perlmutter JS, Mink JW (2006). Deep brain stimulation. Annu Rev Neurosci.

[B44] Henning J, Koczan D, Glass A, Karopka T, Pahnke J, Rolfs A, Benecke R, Gimsa U (2007). Deep brain stimulation in a rat model modulates TH, CaMKIIa and Homer1 gene expression. Eur J Neurosci.

[B45] Elowitz M, Leibler S (2000). A synthetic oscillatory network of transcriptionnal regulators. Nature.

