# Supplementary material for: Simple molecular networks that respond optimally to time-periodic stimulation
Source: BMC Syst Biol. 2009 Mar 3;3:29. doi: 10.1186/1752-0509-3-29 (PMC2666635; doi:10.1186/1752-0509-3-29)
Supplement: Additional File 1 — Supplementary Information. Additional details are given about the mathematical analysis of the periodic activation of the IFFL network by a train of square pulses and about a comparison of our study with the frequency coding analysis performed by Goldbeter et al. in [3]. [file 1752-0509-3-29-S1.pdf]

1 Periodic stimulations of the Incoherent Feedforward Loop network

In this Additional file, we give more details about the mathematical analysis of the periodic activation of the IFFL network by a train of square pulses [cf. eqs. (4)-(5) in the main text]:

$$\dot{Y} = \beta\, S(t) - \alpha Y \tag{1}$$
$$\dot{Z} = \beta\, S(t)\, H(\theta - Y) - \alpha Z \tag{2}$$

The activation of this differential system by a T-periodic train of square pulses $S(t)$ gives rise to periodic solutions for $Y(t)$ and $Z(t)$ having the form of piecewise exponential functions of the time. The periodic evolution of $Y(t)$ can be analytically computed. In particular, eqs. (2) in the main text give analytical expressions for the maximal and minimal values of Y, denoted by Y_{max} and Y_{min}, as well as for $\langle Y \rangle_T$ denoting the mean value of Y averaged over one period T.

The rate of synthesis of Z is ruled by the regulation function $S(t)\, H(\theta - Y(t))$. This function has again the form of a square wave of period T, but now with a shorter duration of the on-phase, denoted by τ'. Therefore, the behaviour of $Z(t)$ has again the same evolution than a simple regulation but now stimulated by a periodic train of pulses with temporal pattern $(\tau', T - \tau')$, representing respectively the pulse and of the inter-pulse intervals. Consequently, the observables Z_{max}, Z_{min} and $\langle Z \rangle_T$ can be computed as for $Y(t)$, but replacing τ by τ' and σ by $T - \tau'$ in the eqs. 2 of the main text.

Figures 1(a)-(b) show the profiles of Z_{max} and $\langle Z \rangle_T$ in function of σ for a fixed pulse duration τ. Two cases must be distinguished, according to the size of the pulse duration τ compared with the "proper" pulse duration τ_θ. This time interval, defined also in the main text, characterises the time needed by Y to reach its repression threshold θ after the onset of $S(t)$. It can be simply computed as (with $\kappa = \dfrac{\beta}{\alpha}$):

$$\tau_\theta = -\frac{1}{\alpha} \log(1 - \theta/\kappa) \tag{3}$$

Let us consider a periodic train of pulses with a fix duration τ, and analyse the behaviour of $\langle Z \rangle_T$ in function of the inter-pulse interval σ. Figures 1(a)-(b) show that when σ is close to zero, the lowest value of the repressor Y_{min} is above θ, and so Y represses Z permanently. This situation holds for a non-zero range of σ, in fact as long as Y_{min} keeps higher than θ. Let us denote by σ_0 the first inter-pulse interval for which $Y_{\text{min}} = \theta$. This can be explicitly be computed as:

$$\sigma_0 = \frac{1}{\alpha} \log\left(1 + \frac{\kappa}{\theta}(e^{\alpha\tau} - 1)\right) - \tau \tag{4}$$

For larger inter-pulse intervals, $\sigma > \sigma_0$, the repressor Y oscillates with minimum values lower than θ and gives rise to small intervals of time, say τ', where the regulatory function of Z is turned on. Therefore Z starts also to oscillate, with maximum, minimum and mean values increasing in function of σ. In fact the duration of this new on-phase, during which Z is activated, can be computed by considering the time taken by Y to pass from Y_{min} to θ, i.e:

$$\tau' = \frac{1}{\alpha} \log\left(\frac{\kappa - Y_{\text{min}}}{\kappa - \theta}\right) \qquad \text{(with } Y_{\text{min}} < \theta) \tag{5}$$

This result makes sense only if $\tau' > 0$, i.e. $Y_{\rm min} < \theta$, otherwise we define $\tau' = 0$. On the other hand, from eq. (2) above, it is clear that the duration of the activation of $Z(t)$ lasts at most τ, so $\tau' \leq \tau$. Using this condition, the expression of $Y_{\rm min}$ as a function of (τ, σ) can be substituted in the last equation, to obtain:

$$\tau' = \min\left(\tau, \frac{1}{\alpha}\log\left(\frac{1-e^{-\alpha\sigma}}{(1-e^{-\alpha(\tau+\sigma)})(1-\theta/\kappa)}\right)\right) \tag{6}$$

The case of long pulses, $\tau > \tau_\theta$

Figure 1(a) illustrates this case. Here $Y_{\rm max}$ stays always above its repression threshold θ, and $\tau' < \tau$ is always true. In this case $Z_{\rm max}$ and $\langle Z\rangle_T$ are computed in function of (τ, σ) by using eqs.(2) of the main text, giving:

$$Z_{\rm max} = \kappa(\frac{1}{1-e^{-\alpha(\tau+\sigma)}} - (1-\frac{\theta}{\kappa})\frac{1}{1-e^{-\alpha\sigma}}) \tag{7}$$

$$\langle Z\rangle_T = \frac{\kappa}{\alpha(\tau+\sigma)}\log\left(\frac{1-e^{-\alpha\sigma}}{(1-e^{-\alpha(\tau+\sigma)})(1-\theta/\kappa)}\right) \tag{8}$$

with again the assumption $\sigma \geq \sigma_0$ (eq.(4)), otherwise $Z_{\rm max} = \langle Z\rangle_T = 0$. In this case $Z_{\rm max}$ is a monotonous and increasing function of σ. On the contrary, $\langle Z\rangle_T$ has a maximum which in principle can be calculated by differentiation of $\langle Z\rangle_T$ with respect to σ.

The case of short pulses, $\tau < \tau_\theta$

The second case, illustrated in Fig.1(b), concerns pulse durations smaller than τ_θ Then there is a time lapse σ_1 between the pulses for which the maximal value of the repressor $Y_{\rm max}$ is below its repression threshold θ for all $\sigma > \sigma_1$. This inter-pulse interval can be computed by the condition $Y_{\rm max}(\sigma_1) = \theta$, which is equivalent to:

$$\sigma_1 = -\frac{1}{\alpha}\log\left(1 - \frac{\kappa}{\theta}(1-e^{-\alpha\tau})\right) - \tau \tag{9}$$

Consequently for $\sigma > \sigma_1$ the repressor is no longer functional and only the regulation by the activator signal S has an effect. Thus for $\sigma > \sigma_1$, one reaches the value $\tau' = \tau$ in eq.(6), and therefore $Z_{\rm max} = Y_{\rm max}$, and $\langle Z\rangle_T = \langle Y\rangle_T$, as illustrated in Fig.1(b). In this situation a maximal value appears also for the maximum $Z_{\rm max}$ when the inter-pulse interval takes the value $\sigma = \sigma_1$. The same interval of time gives an optimal value for $\langle Z\rangle_T$ $\langle Z\rangle_T$ is computed in function of (τ, σ) by using eqs.(2) of the main text, giving:

$$\langle Z\rangle_T = \frac{min\left(max\left(0, \log\left(\frac{1-e^{-\alpha\sigma}}{(1-e^{-\alpha(\tau+\sigma)})(1-\theta/\kappa)}\right)\right), \sigma\right)}{(\tau+\sigma)} \tag{10}$$

(a)

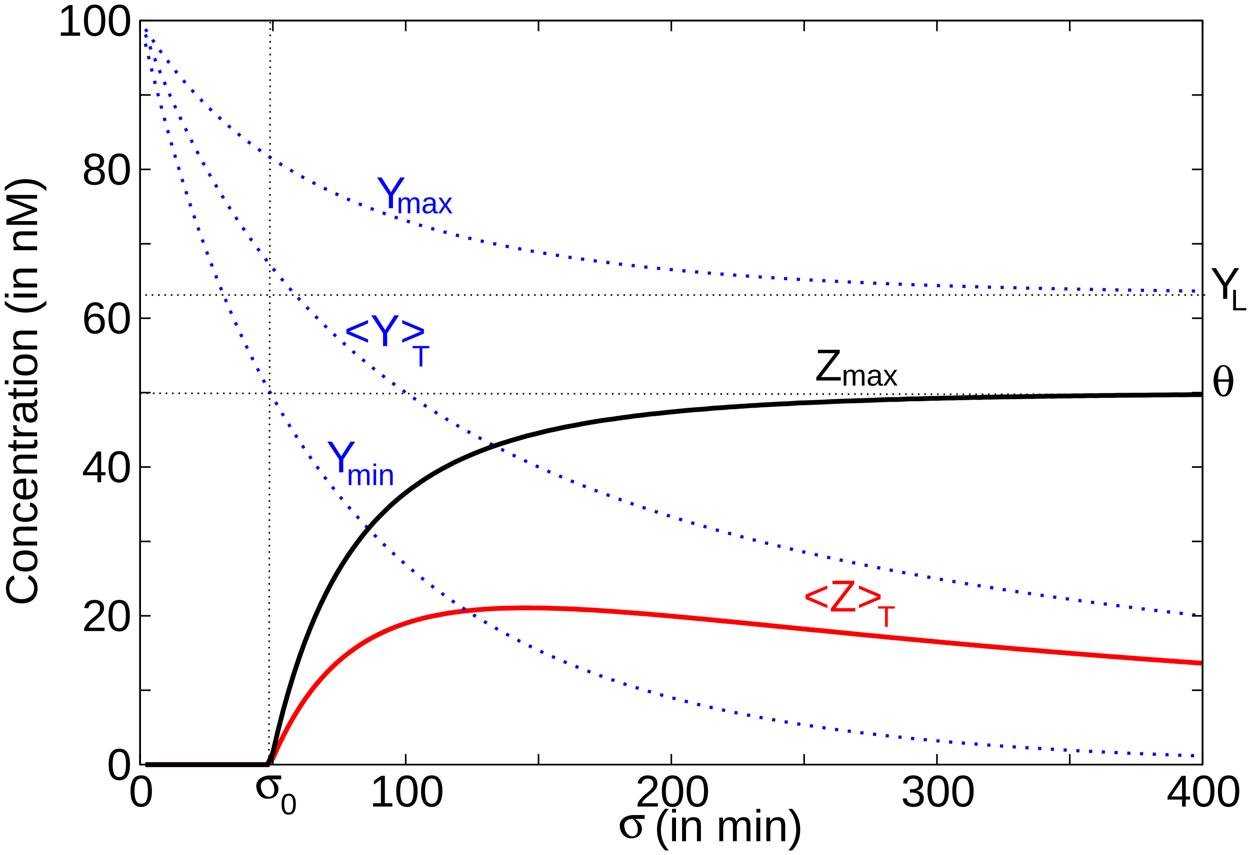

100
80
60
40
20
0
Concentration (in nM)
Y_max
Y_L
<Y>_T
Z_max
θ
Y_min
<Z>_T
0
σ_0
100
200
300
400
σ (in min)

(b)

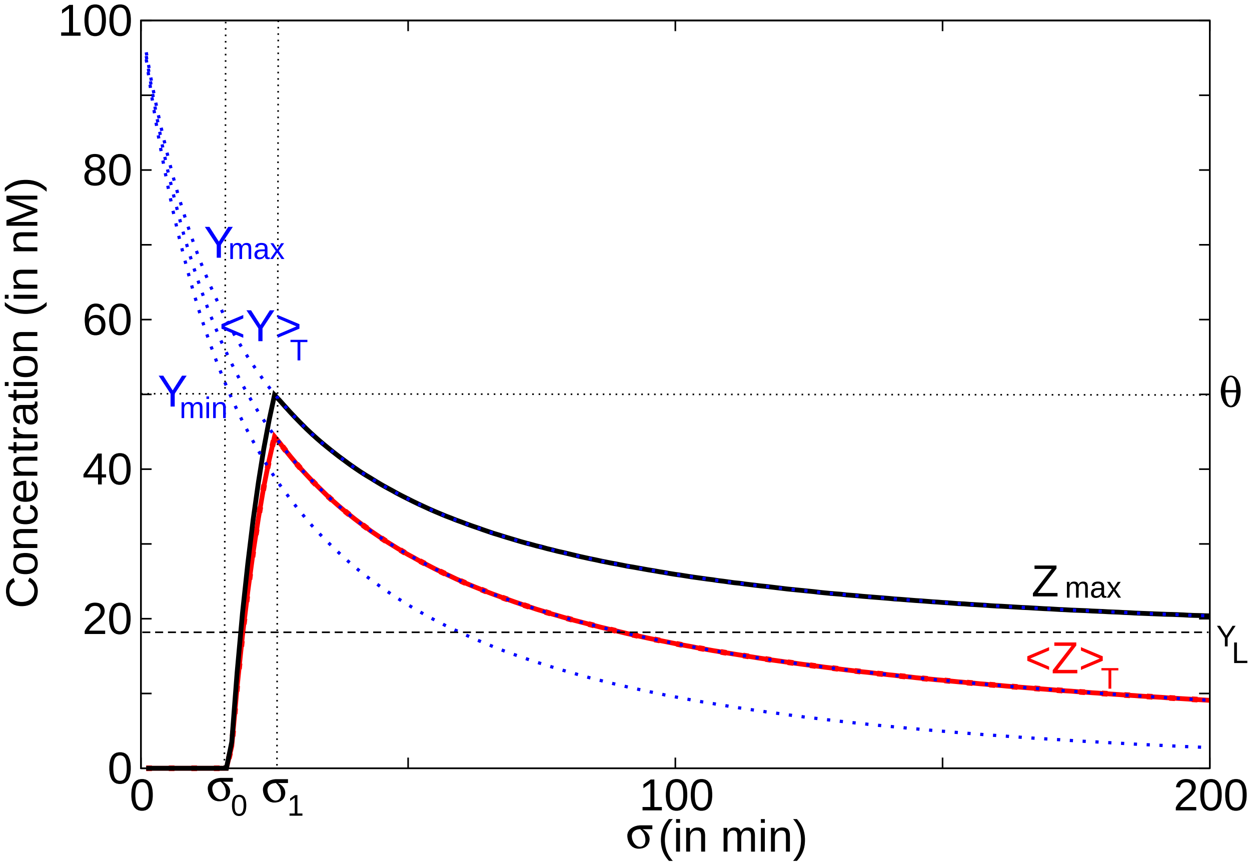

Figure 1: **Response of the IFFL to a periodic stimulation in function of σ.** $< Z >_T$ the mean concentration averaged over one period T and Z_{max} the maximum value reached by Z are represented in function of the inter-pulse interval σ with a fixed pulse duration τ for an IFFL motif stimulated by a pulsatile signal. The evolution of the extreme values of the repressor Y are also shown: Y_{max} the maximum value reached by the protein, $< Y >_T$ the mean concentration of the protein averaged over one period T and Y_{min} the minimal value reached by the protein. $\kappa = \frac{\beta}{\alpha}$ (dotted line) represents the stationary state that the protein Y would attain if the stimulation was constant and Y_L (dashed line) is the asymptotic value reached by Y_{max} when the inter-pulse interval σ becomes very large. σ_0 is the minimal inter-pulse interval for the system to respond and the inter-pulse interval σ_1 gives the optimum average response for the system. The numerical simulation was done with the following parameters: $\alpha = 0.01\ min^{-1}$, $\beta = 1\ nM.min^{-1}$, $\theta = 50nM$, $\tau_\theta = 70$ min. (a) with $\tau > \tau_\theta$, $\tau = 100\ min$, we have $\sigma_1 \sim 145$ min. (b) with $\tau < \tau_\theta$, $\tau = 20\ min$, we have $\sigma_1 \sim 25$ min.

Response of the Incoherent Feedforward Loop with the use of Hill functions

In the main text, we used the logic approximation to represent genetic regulations because it allowed us to obtain some analytical estimations for $Z_{\max}$ and $\langle Z \rangle_T$. However, such approximation is not appropriate for many biological systems which display graded responses and do not behave in an "all-or-none" way. We show in this section two numerical simulations of the Incoherent Feedforward Loop (IFFL) with the use of classical Hill functions. The equations of this modified model are the following:

$$\begin{cases} \dot{Y} = \beta_1 \frac{X^n}{\theta_1^n + X^n} - \alpha Y \\ \dot{Z} = \beta_2 \frac{X^n}{\theta_1^n + X^n} \frac{\theta_2^n}{\theta_2^n + Y^n} - \alpha Z \end{cases} \tag{11}$$

where $X(t)$ is an external square signal. The first figure (Fig.2(a)) shows the response with the Hill exponent $n = 2$, which is common in transcriptional regulations where the proteins often dimerise before interacting with the binding site. This graph, which is presented with a logarithmic scale, clearly shows that this system significantly responds only for specific off-phases durations. A similar example using a Hill exponent $n = 4$ is presented in Fig.2(b). The result is close to the response curve discussed in the the main text (Fig.5(b)). Again the durations between the periodic pulses must have a minimal value to induce a significant response in the system.

2 Comparison with the model of frequency coding proposed by Goldbeter et al. [1]

In the main text, we showed that a square signal can increase the average response of a signaling cycle modeled with linear equations. Here we report that this result is also valid in the case of nonlinear equations proposed by Goldbeter et al. in [2] [1]. This model was studied to demonstrate the possibility of a frequency encoding phenomenon by intracellular Ca^{2+} oscillations. A substrate protein W is phophorylated thanks to a kinase that is sensitive to Ca^{2+} oscillations. The fraction of W in the phosphorylated form is denoted W^*. The equations of this model are the following:

$$\dot{W^*} = (\nu_P / W_T) \left[\nu_K / \nu_P \frac{1 - W^*}{K_1 + 1 - W^*} - \frac{W^*}{K_2 + W^*} \right] \tag{12}$$

with :

$$\nu_K = V_{MK} \frac{Z}{K_a + Z} \tag{13}$$

We have performed numerical simulations of this system with a square signal $Z(t)$, where the duration σ of the off-phase is varied. By increasing the inter-pulse interval, the average response of the fraction of phosphorylated protein W^* decreases, which agrees with the prediction found in [1], when the frequency is decreased.

Our study corroborates a conclusion which was already pointed out in [3], namely that what is crucial in the oscillations encoding is not only the frequency but also the form of the signal and in particular the ratio τ/σ of the on/off phases. As a matter of fact in the case of Ca^{2+} oscillations,

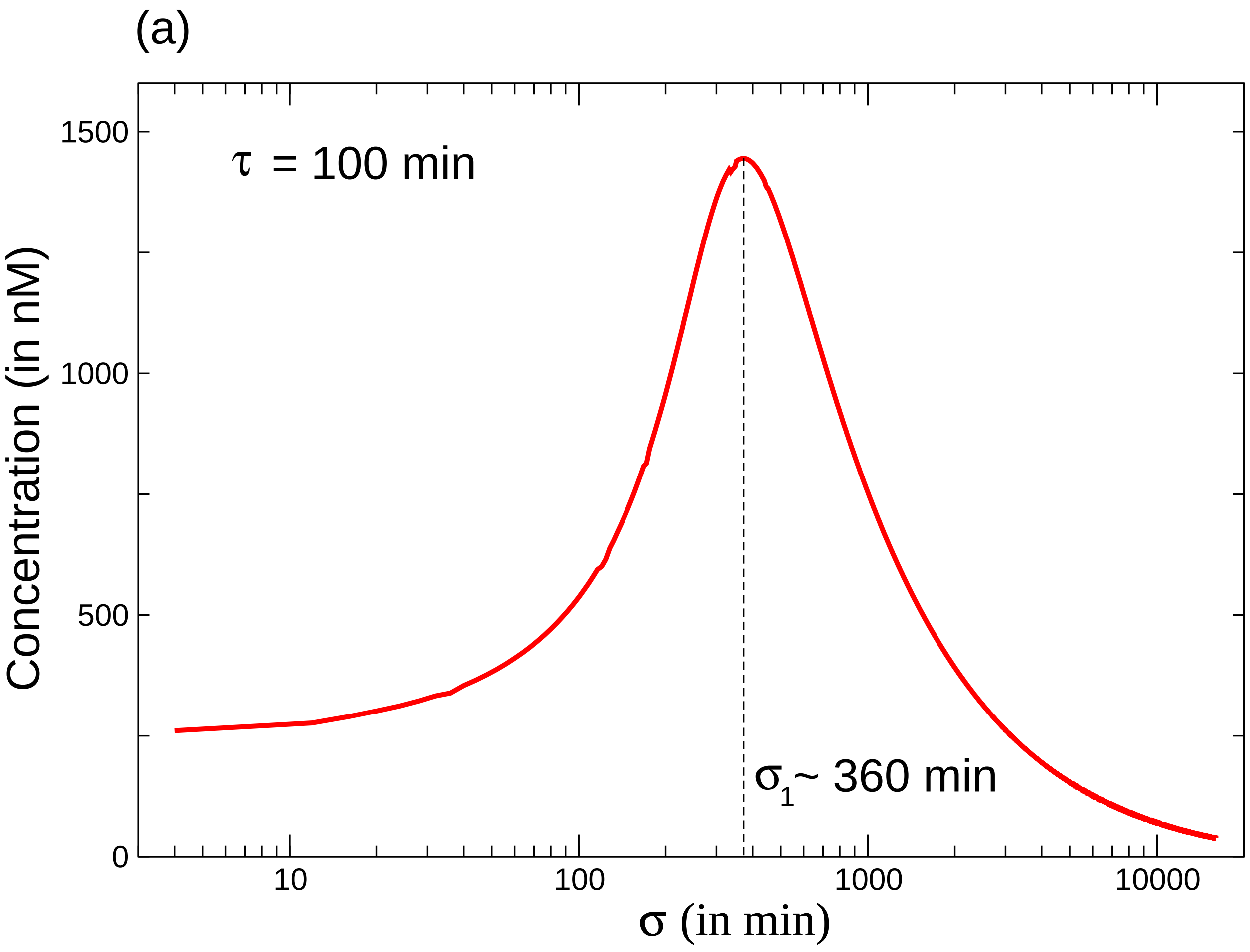

(a)
τ = 100 min
σ1~ 360 min
Concentration (in nM)
σ (in min)
0
500
1000
1500
10
100
1000
10000

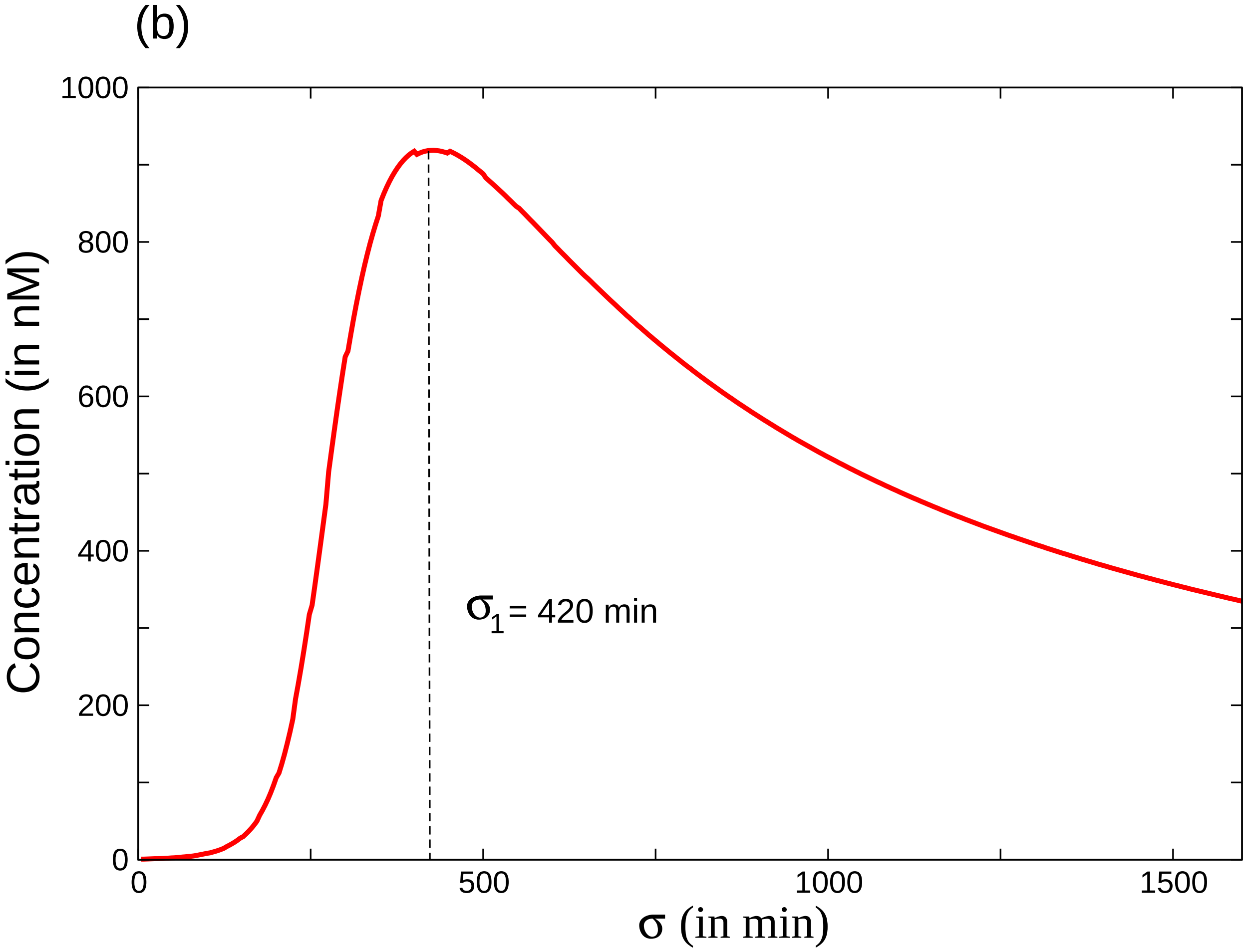

Figure 2: **Response of the IFFL to a periodic stimulation in function of σ simulated with the use of classical Hill function.** $< Z >_T$ the mean concentration averaged over one period T is represented in function of the inter-pulse interval σ with a fixed pulse duration τ for an IFFL motif stimulated by a pulsatile signal. The numerical simulation was done with the equations (11) of Additional material and the following parameters: $\alpha = 0.01\ min^{-1}$, $\beta_1 = 10\ nM.min^{-1}$, $\beta_2 = 1000\ nM.min^{-1}$, $\theta_1 = 50$ nM, $\theta_2 = 50$ nM, $\tau = 100$ min. (a) with a Hill coefficient n = 2, we have $\sigma_1 \sim 360$ min. (b) with a Hill coefficient n = 4, we have $\sigma_1 \sim 420$ min.

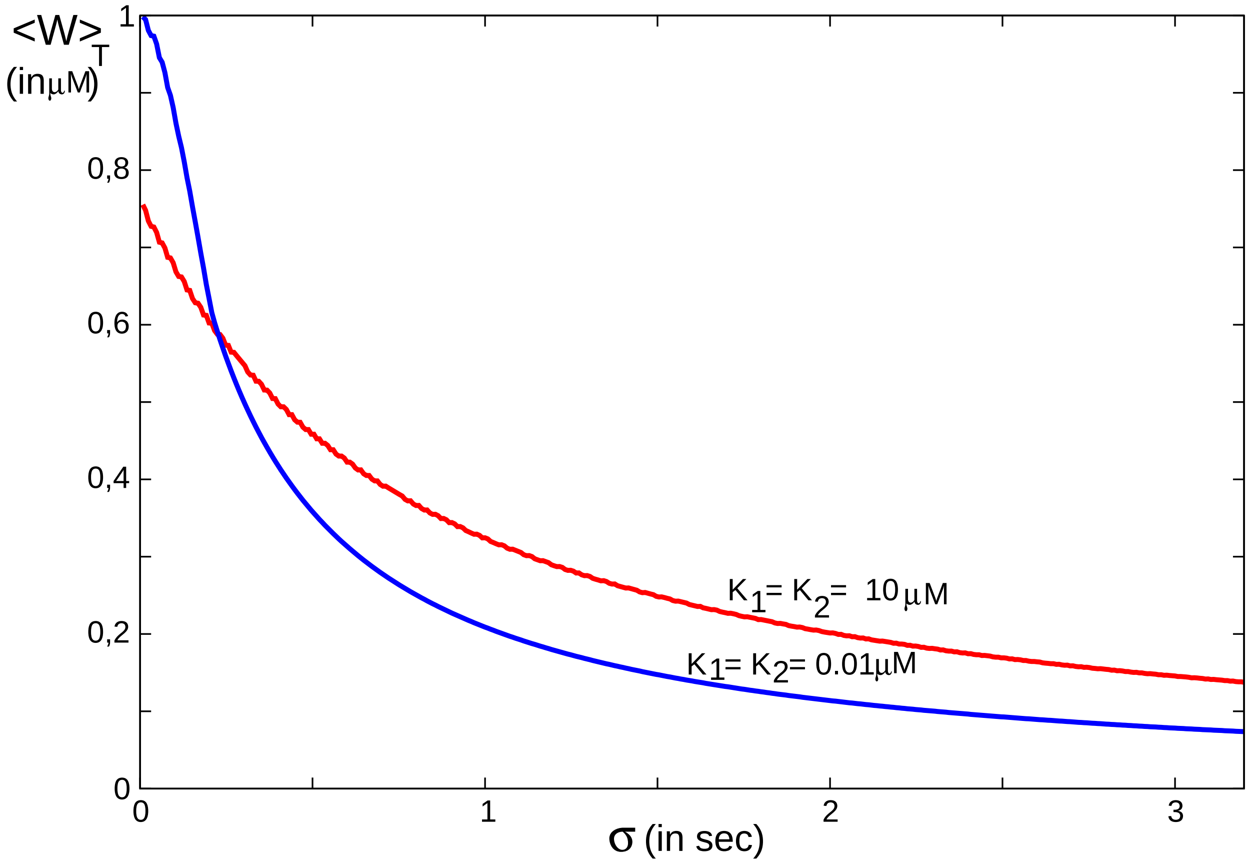

Figure 3: **Average value of the fraction of the phosphorylated protein $< W^* >$ in response of a pulsatile signal for two different values of the Michaelis constants K_1 and K_2.** This graph shows the average value of the fraction of the phosphorylated protein $< W^* >$ over one period when the system of equ (12-13) is submitted to a pulsatile signal with an on phase of duration τ and off duration σ. The simulation was done with the same parameters of [1]: $\nu_P = 5$ $\mu M.s^{-1}$, $V_{MK} = 40$ $\mu M.s^{-1}$, $K_a = 2.5$, $W_T = 1$ μM and with $K_1 = K_2 = 0.01$ $\mu M.s^{-1}$ or $K_1 = K_2 = 10$ $\mu M.s^{-1}$. The frequency encoding is more efficient in the case of small Michaelis-Menten constants.

the level of the stimulus mainly varies the off phase between the Ca^{2+} spikes but the latter have roughly a constant duration. For example in [1], the Ca^{2+} oscillations could be roughly modelled by square oscillations with $\tau \sim 0.2$ sec and σ varying from 0 to 3 sec. We find that the coding is indeed allowed in this range of pulse patterns. Furthermore, as shown in Fig.3, we retrieve the property that the frequency coding should be more efficient when the cycle is in the "zero-order ultrasensivity" regime (small Michaelis Menten constants) as compared with the linear regime.

References

[1] Goldbeter A, Dupont G, Berridge MJ: **Minimal model for signal-induced Ca2+ oscillations and for their frequency encoding through protein phosphorylation.** *Proc Natl*

Acad Sci U S A 1990, **87**(4):1461–1465.

[2] Dupont G, Goldbeter A: **Protein phosphorylation driven by intracellular calcium oscillations: a kinetic analysis.** *Biophys Chem* 1992, **42**(3):257–270.

[3] Li Y, Goldbeter A: **Frequency specificity in intercellular communication. Influence of patterns of periodic signaling on target cell responsiveness.** *Biophys J* 1989, **55**:125–145.
